# Skeleton and musculature of the male abdomen in Tanyderidae (Diptera, Nematocera) of the Southern Hemisphere

**DOI:** 10.3897/zookeys.809.29032

**Published:** 2018-12-19

**Authors:** Olga G. Ovtshinnikova, Tatiana V. Galinskaya, Elena D. Lukashevich

**Affiliations:** 1 Zoological Institute, Russian Academy of Sciences, Universitetskaya nab., 1, St. Petersburg 199034, Russia Zoological Institute, Russian Academy of Sciences St. Petersburg Russia; 2 Department of Entomology, Faculty of Biology, Lomonosov Moscow State University, Leninskie gory 1–12, Moscow 119234, Russia Lomonosov Moscow State University Moscow Russia; 3 Scientific-Methodological Department of Entomology, All-Russian Plant Quarantine Center, Pogranichnaya 32, Bykovo, Moscow region 140150, Russia Scientific-Methodological Department of Entomology, All-Russian Plant Quarantine Center Bykovo Russia; 4 Borissiak Paleontological Institute of Russian Academy of Sciences, Profsoyuznaya st., 123, Moscow 117647, Russia Borissiak Paleontological Institute of Russian Academy of Sciences Moscow Russia

**Keywords:** crane flies, rotation, terminalia, genitalia, morphology, *
Araucoderus
*, *
Nothoderus
*

## Abstract

The structure of the male terminalia and their musculature of species of tanyderid genera *Araucoderus* Alexander, 1929 from Chile and *Nothoderus* Alexander, 1927 from Tasmania are examined and compared with each other and with published data on the likely relatives. The overall pattern of male terminalia of both genera is similar to those of most Southern Hemisphere genera, with simple curved gonostyli, lobe-like setose parameres, and setose cerci inconspicuous under the epandrium. Both genera have terminalia similarly rotated by 180° (and 90° as an intermediate stage); rotation may be either clockwise or counterclockwise. However, the similar patterns are realized differently: segment VIII is the decreased and asymmetrical due to completely membranose tergite VIII in *Nothoderus* (the first record of such modification in Tanyderidae), but narrow and symmetrical in *Araucoderus*. Accordingly, pregenital muscles are very different between the genera. Based on localization of muscle attachment sites, the hypandrial origin of the stripe between gonocoxites is shown in both genera, and entire membranization of tergite VIII and partial membranization of hypoproct is shown in *Nothoderus*. Tanyderidae are characterized by highly specialized sclerites and muscles of male terminalia and provide no evidence of relationship with previously studied members of Psychodidae, Blephariceridae and Ptychopteridae.

## Introduction

Tanyderidae, or primitive crane flies, are a small ancient nematocerous family with amphitropical distribution and higher recent diversity in the Southern Hemisphere (Eskov and Lukashevich 2015). Only 18 extinct and extant genera (most of them monotypic) are described in the family, which has been known since the Early Jurassic (Ansorge 1994, Skibińska et al. 2014). In the 19^th^ century tanyderids were described in the family Ptychopteridae, but now the family is usually considered to be close to Psychodidae (Crampton 1926, Alexander 1927, Shcherbakov et al. 1995, Krzemiński et al. 2013); however, some current authors consider it to be closely related to Ptychopteridae (Hennig 1973, Wood and Borkent 1989, Oosterbroek and Courtney 1995) or Blephariceridae (Lambkin et al. 2013; Ptychopteridae were not included in the analysis). Close relationships between Tanyderidae, Blephariceridae and Psychodidae was recently inferred from DNA phylogeny (Bertone et al. 2008, Wiegmann et al. 2011) and possible closeness of Tanyderidae and Psychodidae was inferred from DNA phylogeny of Psychodidae (Curler and Moulton 2012).

The family is important for understanding of the history of the order, but remains insufficiently studied. Male genitalia of some extant genera have been described or drawn with varying degrees of detail; both genera from the Northern Hemisphere: *Protoplasa* Osten-Sacken, 1860 and *Protanyderus* Handlirsch, 1909; and most of genera from the Southern Hemisphere: *Peringueyomyina* Alexander, 1921, *Mischoderus* Handlirsch, 1909, *Araucoderus* Alexander, 1929, *Eutanyderus* Alexander, 1928, *Nothoderus* Alexander, 1927 and *Neoderus* Alexander, 1927 (Alexander 1927, 1928, Williams 1933, Colless and McAlpine 1970, Savchenko 1971, 1974, 1978, Borkent and Sinclair 2012, Madriz and Courtney 2016, Madriz 2017, Madriz et al. 2018). Only *Peringueyomyina*, *Araucoderus* and *Neoderus* were described in sufficient detail. Musculature of the terminalia has never been examined in the family.

Among the likely relatives, musculature has been examined in the blepharicerid, *Edwardsinagigantea* Zwick, 1977 (Zwick 1977), the ptychopterid, *Ptychopteralacustris* Meigen, 1830 (Just 1973) and the psychodids, *Phlebotomusgarnhami* Heisch, Guiggsberg & Teesdale, 1956 (Just 1973), *P.papatasi* (Scopoli, 1786) (Jobling 1987), *Pericoma* sp. (Hennig 1936) and *Pneumiapalustris* (Meigen, 1804) (as *Pericomapalustris* in Just 1973). All examined psychodids are members of more derived subfamilies (Phlebotominae and Psychodinae), whereas musculature for members of basal subfamilies has not yet been described. Additionally, the data of different authors are usually difficult to compare, because a uniform nomenclature of muscles is lacking; moreover, figures of Jobling (1987) are not accompanied by any descriptions. Therefore, as the first step for further comparison, a table with the presumed homology of musculature among families was compiled (Table 1). Recently a similar attempt of homologization was undertaken for other families of Diptera (Spangenberg et al. 2012).

**Table 1.** Musculature of male terminalia of Tanyderidae, Psychodidae, Blephariceridae and Ptychopteridae.

Taxa	Muscle Groups									
	Abdominal muscles	Pregenital muscles		Tergosternal muscles	Muscles of the hypandrial complex				Muscles of the epandrial complex	
	Muscles of VII sclerite	Hypandrial muscles	Epandrial muscles		Ejaculator muscles	Aedeagal muscles	Gonocoxal muscles	Gonostylar muscles	Muscles of hypoproct	Muscles of cercus
** Tanyderidae **										
*Araucoderusgloriosus* (present study)	Paired asymmetrical *ISM7*; Paired asymmetrical *ITM7*	Paired asymmetrical *M18*	Paired asymmetrical *M19*	*M5*	*M23*; *M30*; *M31*	*M1+2*	not found	*M27*; *M28*	*M3*	*M7*
*Nothoderusaustraliensis* (present study)	Paired asymmetrical *ISM7*; Unpaired *ITM7*	Unpaired *M18*	not found	*M5*	*M23*; *M30*; *M31*	*M1+2*	not found	*M27*; *M28*	*M3^1–2^*	*M7*
** Psychodidae **										
*Pericomasp.* (after Hennig 1936) *Pneumiapalustris* (after Just 1973)	Paired slightly asymmetrical *ISM7* (*VLM1*, *VLM2* sensu Just 1973); Paired slightly asymmetrical *ITM7* (*DLM1*, *DLM2* sensu Just 1973)	*M18* (*VLM1a*, *VLM1b* sensu Just 1973)	*M19* (*JM8–9* sensu Hennig 1936; *DLM1*, *VLM2* sensu Just 1973)	*M5^1–2^* (*M6*, *M6a* sensu Hennig 1936; *M7*, *M9* sensu Just 1973)	*M30* (*MS1*, *MS2* sensu Hennig 1936; *M4* sensu Just 1973); *M31* (*MS3* sensu Hennig 1936; *M5* sensu Just 1973)	*M1* (MS4 sensu Hennig 1936; *M6* sensu Just 1973); *M2* (*M4* sensu Hennig 1936; *M1* sensu Just 1973)	not found	*M27* (*M3* sensu Hennig 1936; *M2* sensu Just 1973); *M28* (*M5* sensu Hennig 1936; *M3* sensu Just 1973)	*M3* (*M1* sensu Hennig 1936; *M8* sensu Just 1973)	*M7* (*M2a*, *M2b* sensu Hennig 1936; *M10* sensu Just 1973)
*Phlebotomusgarnhami* (after Just 1973)	Paired asymmetrical *ISM7* (*VLM* sensu Just 1973); Paired asymmetrical *ITM7* (*DLM* sensu Just 1973)	*M18* (*VLM* sensu Just 1973)	*M19^1–2^* (*DLM^1–2^*sensu Just 1973)	not found	? *M31^1–2^* (*M5, M6* sensu Just 1973)	*M1* (*M4* sensu Just 1973)?; *M2* (*M1* sensu Just 1973)	not found	*M27* (*M2*, *M2^1^* sensu Just 1973); *M28* (*M3*, *M3^1^* sensu Just 1973)	*M3* (*M7* sensu Just 1973)	*M7* (*M8* sensu Just 1973)
** Blephariceridae **										
*Edwardsinagigantea* (after Zwick 1977)	not examined	*M18* (described without numbering Zwick 1977: p.8 (1))	*M19* (described without numbering Zwick 1977: p.8 (1))	*M5^1–2^* (described without numbering Zwick 1977: p.8 (2))	? *M30* (*M7* sensu Zwick 1977); *M31^1–2^* (*M5*, *M6* sensu Zwick 1977)	*M1* (*M1* sensu Zwick 1977); *M2* (*M2* sensu Zwick 1977)	not examined	*M27* (*M3* sensu Zwick 1977); *M28* (*M4* sensu Zwick 1977)	*M3* (described without numbering Zwick 1977: p.8 (2))	*M7* (described without numbering Zwick 1977: p.8 (2))
** Ptychopteridae **										
*Ptychopteralacustris* (after Just 1973)	Paired symmetrical *ISM7* (*VLM* sensu Just, 1973); Paired symmetrical *ITM7* (*DLM* sensu Just, 1973)	Paired symmetrical *M18* (*VLM2* sensu Just, 1973)	Paired symmetrical *M19* (*DLM2* sensu Just, 1973)	*M5* (*M1* sensu Just, 1973)	*M31* (*M11* sensu Just, 1973)	*M1^1–4^* (*M3*, *M8*, *M9*, *M7* sensu Just, 1973); *M2^1–2^* (*M6*, *M10* sensu Just, 1973)	*M33* (*M2*, sensu Just, 1973);	*M27* (*M4* sensu Just, 1973); *M28* (*M5* sensu Just, 1973)	*M3^1–3^* (*M12*, *M13*, *M14* sensu Just, 1973)	*M7^1–2^* (*M15*, *M16* sensu Just, 1973)

Study of the musculature is helpful not only for specifying the functions of genital sclerites, but also for revealing the homology of some poorly traced structures (Ovtshinnikova and Yeates 1998, Ovtshinnikova and Galinskaya 2016b, 2017, Galinskaya et al. 2018). Based on morphogenetical regularities formulated by Matsuda (1976) and verified by Ovtshinnikova (1989) and Friedrich and Beutel (2008), characters associated with muscles are confirmed to be more stable than those associated with sclerites and therefore can be used successfully in phylogenetic studies; morphological series of different species are especially productive for such studies.

The purpose of this study was to investigate the skeleton and musculature of the male genitalia of two genera of Tanyderidae from the Southern Hemisphere. The muscles and sclerites of the male abdomen of *Nothoderusaustraliensis* (Alexander, 1922) from Tasmania and the muscles of the male abdomen of *Araucoderusgloriosus* (Alexander, 1920) from Chile are described for the first time.

## Material and methods

This study is based on males of Tanyderidae collected in *Nothofagus*-dominated forests in Chile and Tasmania by D. Shcherbakov and E. Lukashevich in 2014 and 2015. Specimens studied herein will be deposited in the Zoological Institute RAS, St-Petersburg, Russia. Additional specimens are deposited in the National Museum of Natural History, Santiago, Chile (*Araucoderus*) and Tasmanian Museum and Art Gallery, Hobart, Australia (*Nothoderus*).

Scanning electron micrographs of uncoated and coated males were taken with a Tescan Vega microscope using backscattered electron (BSE) and secondary electron (SE) detectors.

The terminology of the male genital sclerites mainly follows Cumming and Wood (2009) with additions on structure of the parameres made by Madriz and Courtney (2016). Description of sclerites of *Nothoderusaustraliensis* in this study is modeled on the description of *Araucoderusgloriosus* from the study of Madriz and Courtney (2016).

The muscular systems of male genitalia were studied by manually dissecting the material (preserved fresh in 70% alcohol) with microknives in water under a Leica MZ9^5^ stereomicroscope. The pictures were taken using the image capture function of the Leica MZ9^5^ trinocular head and subsequently processed. The male terminalia muscles were classified into several groups: muscles of the epandrial complex, muscles of the hypandrial complex, tergosternal muscles, and pregenital muscles. The muscles were numbered according to the classification previously accepted by Ovtshinnikova with the following modifications *M21* = *M3*, *M29* = *M7* and *M32* = *M23* based on homologization of muscles (Ovtshinnikova 2000).

List of abbreviations: *aed* – aedeagus; *cerc* – cercus; *ej apod*– ejaculatory apodeme; *ep* – epandrium; *goncx* – gonocoxite; *gonst* – gonostylus; *hypd* – hypandrium; *hypp* – hypoproct; *ISM* – abdominal intersegmental sternal muscles; *ITM* – abdominal intersegmental tergal muscles; *lepr* – lateral ejaculatory process; *M1*–*M33* – pregenital and genital muscles; *pm db* – dorsal bridge of paramere; *pm dme* – dorsomedial element of paramere; *pm gbl* – paramere lobe at gonocoxite base; *pm lme* – lateromedial element of paramere; *spm sac* – sperm sac; *st* – sternite; *tes* – testis; *th* – thorax; *tg* – tergite; *TSM* – abdominal tergosternal muscles.

## Results

### Family Tanyderidae Osten-Sacken, 1880

#### Subfamily Tanyderinae Osten-Sacken, 1880

The subfamily includes nine extant and five extinct genera with relatively short gonopods. Musculature of male terminalia is here examined for only two members of the subfamily.

Tanyderidae are characterized by abdominal segments with intersegmental tergal (*ITM*), intersegmental sternal (*ISM*) and tergosternal (*TSM*) muscles and by pregenital muscles (*M18*, *M19*). Male genital muscles of Tanyderidae are classified into several groups: tergosternal muscles (*M5*); muscles of the hypandrial complex (3 pairs of ejaculator muscles *M23*, *M30*, *M31*; aedeagal muscle *M1+2*; 2 pairs of gonostylar muscles *M27*, *M28*); muscles of the epandrial complex (muscles *M3*, connecting epandrium with hypoproct (=X sternite); muscles *M7*, connecting hypoproct with cerci).

##### Araucoderus gloriosus

Taxon classificationAnimaliaDipteraTanyderidae

(Alexander, 1920)

**Figure 1.**
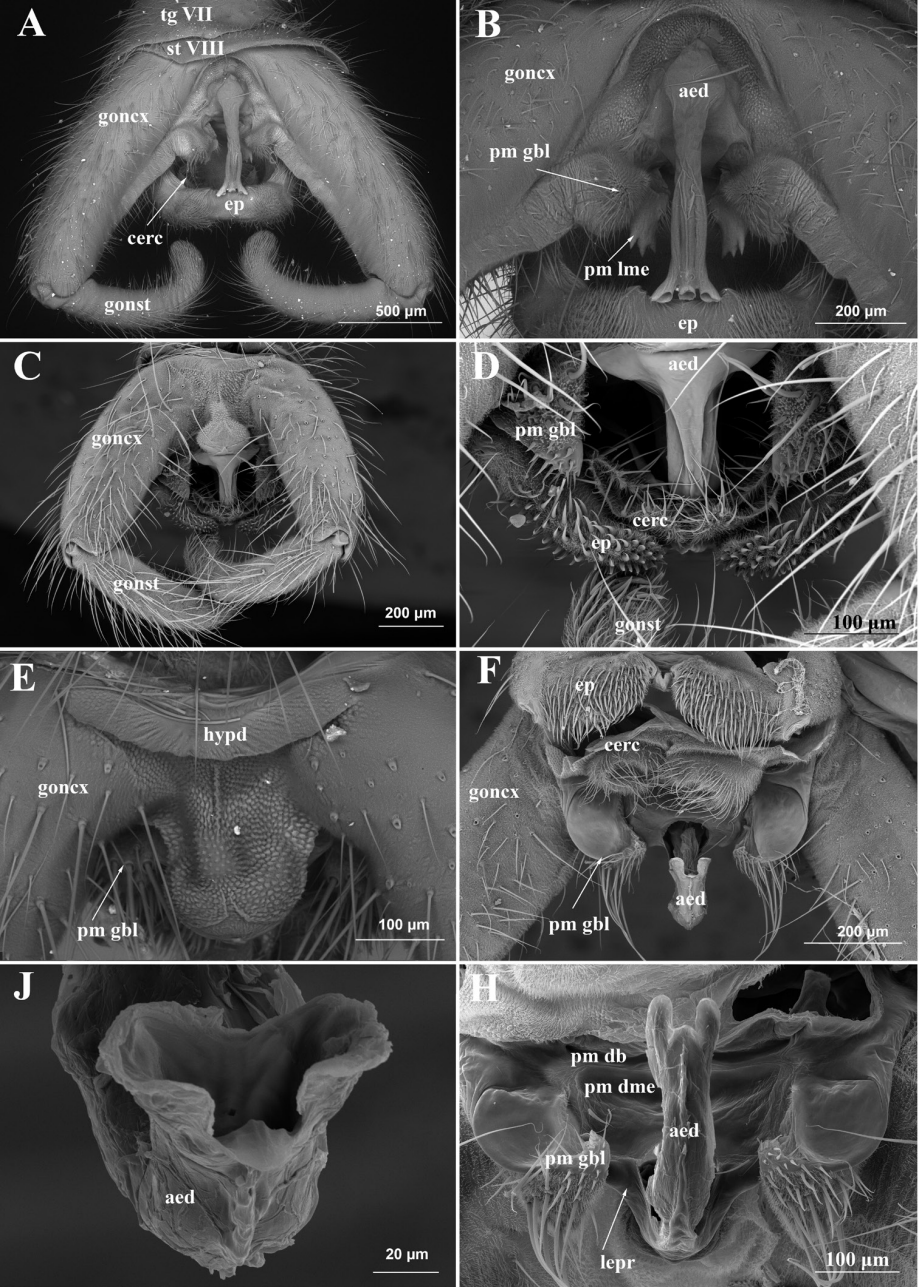
SEM images of male genitalia of Tanyderidae. **A–B***Araucoderusgloriosus* (uncoated, BSE; Chile, Alerce Andino), dorsal view **C–H***Nothoderusaustraliensis***C–E** dorsal view (uncoated, BSE; Tasmania, Mystery Creek Cave) **F–H** ventral view (coated, SE; Tasmania, Lake Saint Clair).

**Figure 2.**
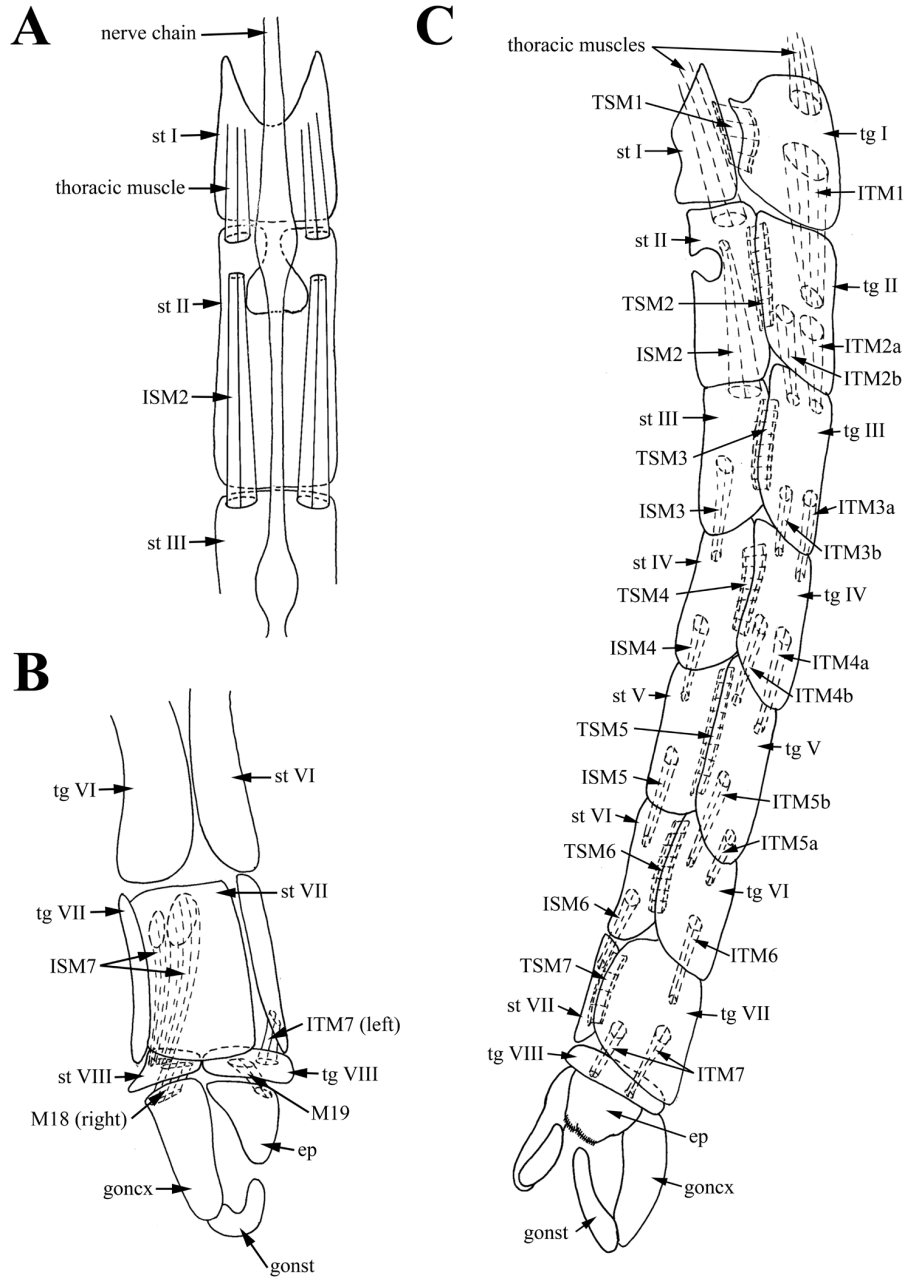
Male of *Araucoderusgloriosus*. **A** sternites, inner view **B** tip of abdomen, right lateral view **C** abdomen, left lateral view.

**Figure 3.**
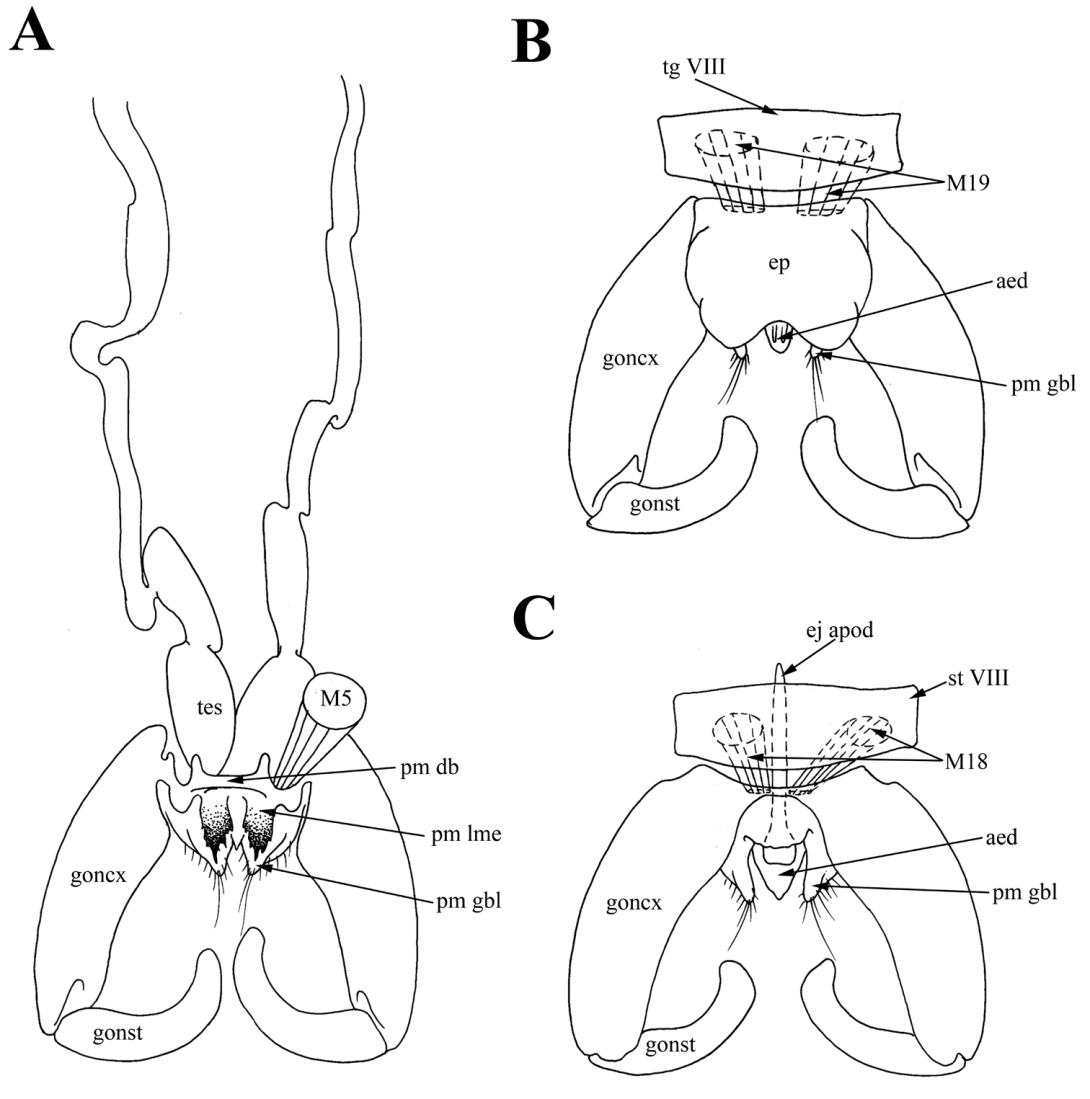
Male of *Araucoderusgloriosus*. **A** genitalia, inner view **B** genitalia, ventral view **C** genitalia, dorsal view.

**Figure 4.**
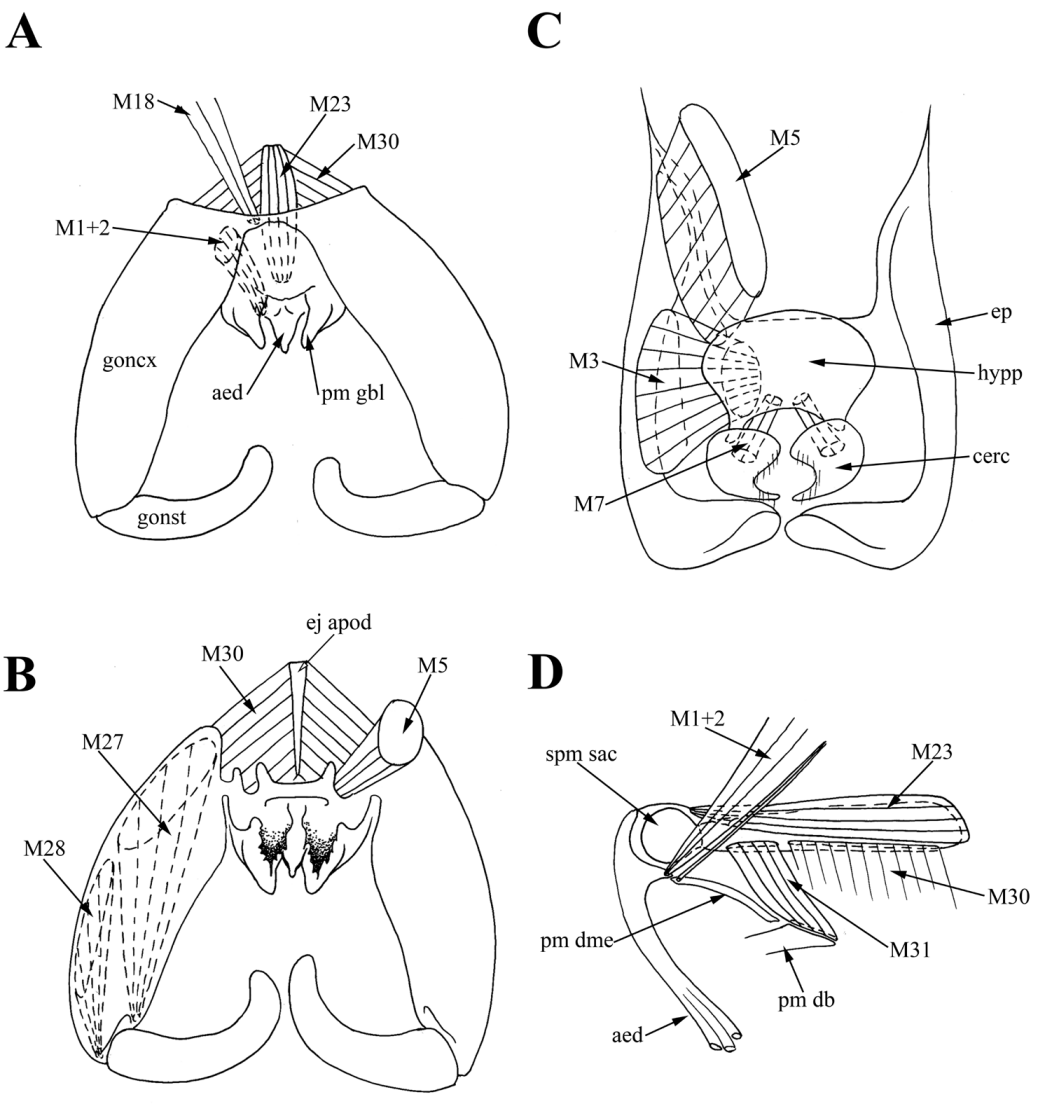
Male of *Araucoderusgloriosus*. **A** genitalia, dorsal view **B** genitalia, inner view **C** epandrium, inner view **D** aedeagal complex, lateral view.

Figures 1A, B , 2 , 3 , 4

###### Material.

Chile, *Nothofagus*-dominated forest, on riparian vegetation. Alerce Andino National Park: Lenca River, 340 m asl (41°30'S, 72°37'W), 6–8.i.2014, 12–18.i.2015, D.E. Shcherbakov, E.D. Lukashevich, 8 males; Puyehue National Park: Anticura River near Anticura Waterfall, 400 m asl (40°40'S, 72°10'W), 14.i.2014, E.D. Lukashevich, 1 male; Chanlefu River at Aguas Calientes, 470 m asl (40°44'S, 72°18'W), 16.i.2014, D.E. Shcherbakov, 1 male; Huerquehue Natianal Park: near Tiquilco Lake, 780 m asl, (39°10'S, 71°44'W), 22.xii.2014, E.D. Lukashevich, 1 male; near La Junta, Rio Palena, 70 m asl, (43°49'S, 72°21'W), 5.i.2015, E.D. Lukashevich, 1 male. The specimens will be deposited in the Zoological Institute RAS, St-Petersburg, Russia.

###### Exoskeleton.

The male terminalia were described in great detail by Madriz and Courtney (2016), so here we describe only the muscles; scanning electron micrographs of genitalia are published for comparison purposes (Figure 1A, B).

###### Musculature.

*Thoracic muscles.* One pair of tergal muscles, connecting thorax and medial part of tergite I; one pair of sternal muscles, connecting thorax and anterolateral margin of sternite II (Figure 2C).

*Abdominal muscles.* One pair of long intersegmental tergal muscles *ITM1* connecting medial part of tergite I and medial part of tergite II (Figure 2C). Segments I–VII with wide, short tergosternal muscles (*TSM1*–*TSM7*) (Figure 2C). One pair of long symmetrical intersegmental sternal muscles *ISM2* passing from anterior third of sternite II to anterior margin of sternite III. Two pairs of long symmetrical intersegmental tergal muscles (*ITM2a,b*) passing from posterior half of tergite II to anterior margin of tergite III; medial ITM2a slightly thinner and longer, lateral *ITM2b* slightly stronger but shorter (Figure 2A, C). Intersegmental tergal and sternal muscles on segments III–V with similar attachment sites as intersegmental muscles of segments II–III (Figure 2C). One pair of intersegmental tergal muscles *ITM6* extending from tergite VI to tergite VII. One pair of intersegmental sternal muscles *ISM6* extending from sternite VI to sternite VII. Intersegmental muscles *ITM6* and *ISM6* thinner than intersegmental muscles of previous segments; posterior sites of attachment of muscles *ITM6* and *ISM6* slightly displaced counterclockwise due to terminalia rotation (Figure 2C). One pair of long asymmetrical muscles *ISM7* extending from middle of anterior part of sternite VII to left anterior margin of sternite VIII; posterior sites of attachment of muscles *ISM7* displaced counterclockwise due to terminalia rotation (Figure 2B). Tergite VIII situated on left side of abdomen. One pair of asymmetrical muscles *ITM7* extending from tergite VII to tergite VIII. Right muscle *ITM7* long, extending from middle of tergite VII to posterior part of tergite VIII. Left muscle *ITM7* short, extending from posterior margin of tergite VII to posterior part of tergite VIII. Posterior sites of attachment of muscles *ITM7* displaced counterclockwise due to terminalia rotation (Figure 2B, C).

*Pregenital muscles.* Paired short asymmetrical muscles *M18*: right muscle *M18* long, connecting middle of right side of sternite VIII to narrow sclerotized stripe or hypandrium between gonocoxite bases; left wide short muscle *M18* connecting middle of left side of sternite VIII to narrow sclerotized stripe between gonocoxite bases (Figures 2B, 3C). Narrow sclerotized stripe between gonocoxite bases interpreted as hypandrium according to attachment sites of muscles *M18*. Paired short, wide and slightly asymmetrical muscles *M19* extending from center of tergite VIII to anterior margin of epandrium (Figure 3B).

*Tergosternal muscles.* Paired, wide symmetrical *M5* connecting anterolateral parts of epandrium to lateral thickenings of dorsal bridge of paramere in the point of connection of *pm db* with gonocoxites (= gonocoxal apodeme) (Figures 3A, 4B, C). Lateral thickenings of dorsal bridge of paramere interpreted as gonocoxites according to attachment sites of muscles *M5*.

*Muscles of the hypandrial complex.* Paired long retractors *M1+2* extending from anterior edges of gonocoxites to aedeagal condyle, and several muscle filaments of *M1+2* extending from anterior edges of gonocoxites to posterior margin of dorsomedial element of paramere *pm dme* (Figure 4A, D). Paired long *M23* extending from posterior edge of ejaculatory apodeme (dorsally) to membrane near aedeagus (Figure 4A, D). Paired wide protractors *M30* extending from anterior half of ejaculatory apodeme (laterally) to anterior margin of gonocoxites (Figure 4A, B, D). Paired retractors *M31* extending from posterior part of ejaculatory apodeme to medial part of dorsal bridge of paramere *pm db* (Figure 4D). Paired long wide *M27* extending from anterior part of gonocoxites to ligament near anterior margin of gonostyli (Figure 4B). Paired long wide *M28* extending from medial part of gonocoxites to condyle of gonostyli (Figure 4B).

*Muscles of the epandrial complex.* Paired short wide *M3* extending from most of inner epandrium surface to hypoproct (X sternite) (Figure 4C). Paired thin *M7* extending from posterolateral parts of hypoproct to cerci (Figure 4C).

##### Nothoderus australiensis

Taxon classificationAnimaliaDipteraTanyderidae

(Alexander, 1922)

**Figure 5.**
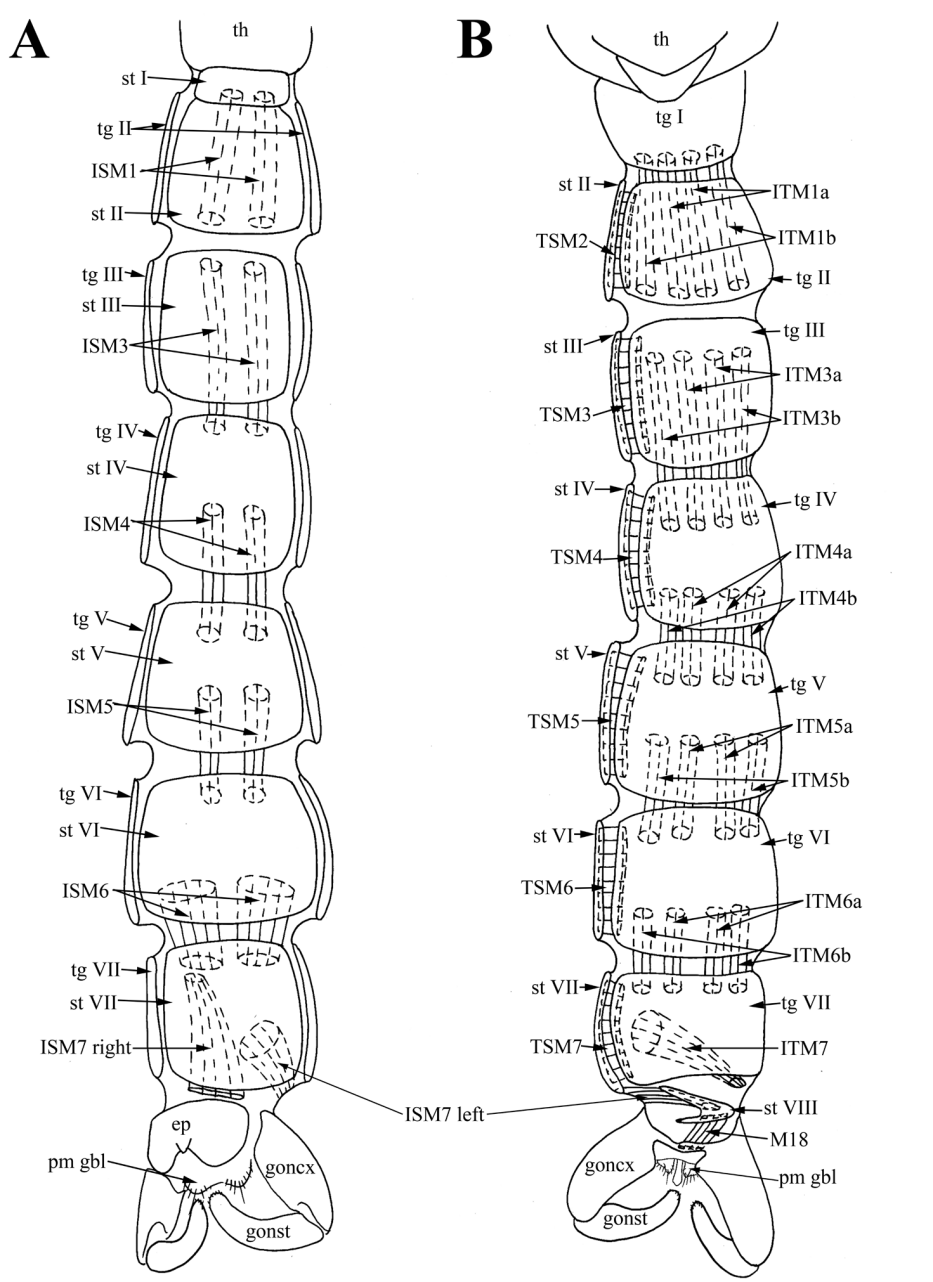
Male of *Nothoderusaustraliensis*. **A** abdomen, ventral view **B** abdomen, dorsal view.

**Figure 6.**
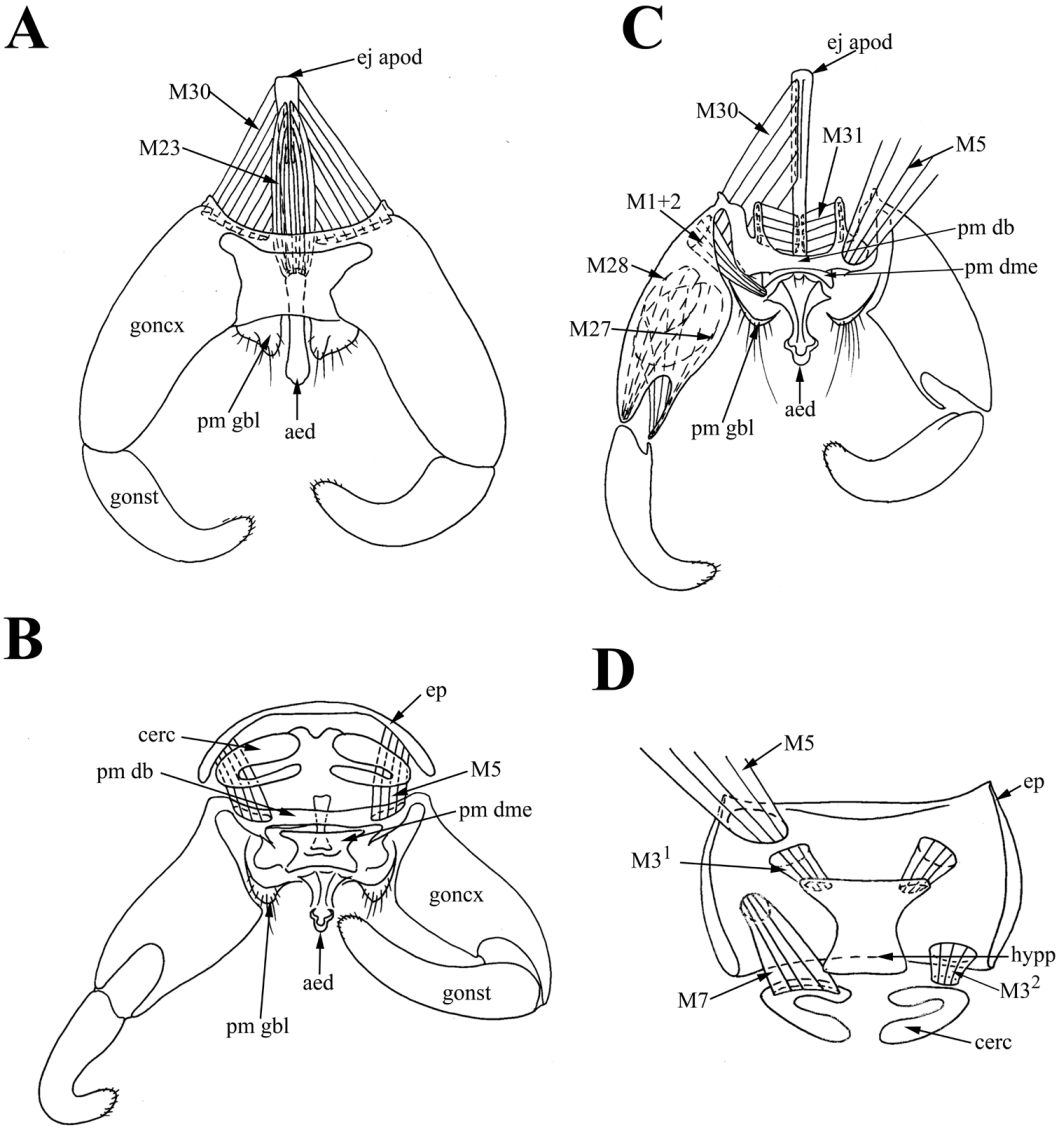
Male of *Nothoderusaustraliensis*. **A** genitalia, dorsal view **B** genitalia, posterior view **C** genitalia, inner view **D** epandrium, inner view.

Figures 1C–H , 5 , 6

###### Material.

Tasmania, *Nothofagus*-dominated forest, on riparian vegetation. Lake Saint Clair National Park (42°6'S, 146°9'E), 7.xii.2015, D.E. Shcherbakov, 2 males; Mystery Creek Cave (43°28'S, 146°51'E), 13.xii.2015, D.E. Shcherbakov, E.D. Lukashevich, 4 males. The specimens will be deposited in the Zoological Institute RAS, St-Petersburg, Russia.

###### Exoskeleton.

Abdomen: tergite I about 0.6 times as long as tergite II, sternite I about 0.3 times as long as sternite II (Figure 5). Segments II–VII well developed. Terminalia with 160–180° rotation of through segments VII–IX, with segment VIII rotated about 80–90°. Segment VIII reduced to one small sclerite situated dorsally (Figure 5B). Gonocoxites and gonostyli pubescent with long setae. Gonocoxites narrowly contiguous at base, divergent from each other at origin, each nearly cylindrical, tapered slightly toward apex (Figures 1C, 5, 6A). Gonostylus cylindrical, about 4/5 length of gonocoxite, slightly tapered at apex, curved medially, with dense macrosetae apically (Figures 1C, D, 5). Apparent hypandrium as narrow sclerotized stripe between gonocoxite bases (Figure 1E), according to muscle attachment sites (see musculature description). Epandrium slightly wider than long, with elongate setae along lateral margins and robust closely set macrosetae on two large convex lobes; tiny bare medial lobe between lateral ones; cercus inconspicuous, unmodified, setose, with semicircular rim (Figures 1D, F, 6B). Hypoproct small, situated ventrad of epandrium (Figure 6E). Paramere subdivided into dorsal bridge (*pm db*), dorsomedial element (*pm dme*) and parameral lobe at gonocoxite base (*pm gbl*) (Figures 1D, F, H, 6B): dorsal bridge arch-shaped, with one pair of long projections anterolaterally; dorsomedial element “I-shaped”, articulated with dorsal bridge basally and with lateral ejaculatory processes of aedeagus apically; parameral lobe at gonocoxite base bare inside and with group of elongated setae among shorter ones outside (Figure 1D, F, H). Ejaculatory apodeme extending anteriorly to segment VII, laterally compressed, clavate at base (Figure 6A, C); sperm sac balloon-like, surrounded by aedeagus posteriorly, attached to ejaculatory apodeme anteriorly; aedeagus relatively short, curved, with single trilobate phallotrema, placed between cerci when at rest (Figure 1F–H).

###### Musculature.

*Abdominal muscles.* Tergosternal muscles *TSM1* of segment I not found. Segments II–VII with one pair of wide short tergosternal muscles (*TSM2*–*TSM7*) (Figure 5B). Two pairs of long symmetrical intersegmental tergal muscles (*ITM1a,b*) extending from posterior part of tergite I to posterior part of tergite II; paired medial *ITM1a* and paired lateral *ITM1b* with same thickness (Figure 5B). One pair of long intersegmental sternal muscles *ISM1* connecting posterior part of sternite I to posterior part of sternite II (Figure 5A). Intersegmental tergal and sternal muscles *ITM2* and *ISM2* connecting segment II and III not found. Intersegmental symmetrical tergal and sternal muscles *ITM3a,b*–*ITM5a,b* and *ISM3*–*ISM5* on segments III–VI with similar attachment sites as intersegmental muscles of segment I–II (Figure 5). Segments II–VII with wide short tergosternal muscles (*TSM2*–*TSM7*) (Figure 5B). Two pairs of intersegmental tergal muscles *ITM6a,b* extending from posterior part of tergite VI to anterior part of tergite VII. One pair of symmetrical wide intersegmental sternal muscles *ISM6* passing from posterior part of sternite VI to anterior part of sternite VII. Posterior sites of attachment of muscles *ITM6a,b* and *ISM6* slightly displaced clockwise (Figure 5). One pair of long asymmetrical muscles *ISM7* right extending from right anterior part of sternite VII to membrane between sternite VII and epandrium. *ISM7* left extending from left medial part of sternite VII to sternite VIII (Figure 5). Unpaired muscle *ITM7* connecting left medial part of tergite VII to membrane between tergite VII and sternite VIII (Figure 5B).

*Pregenital muscles.* Short unpaired muscle *M18* connecting sternite VIII to narrow sclerotized stripe or hypandrium between gonocoxite bases (Figure 5B). Narrow sclerotized stripe between gonocoxite bases interpreted as hypandrium, according to attachment sites of muscles *M18*. Muscles *M19* not found. Segment VIII decreased to one small dorsal sclerite; tergite VIII completely membranous, sternite VIII reduced to narrow sclerite.

*Tergosternal muscles.* Paired, wide symmetrical *M5* connecting anterolateral parts of epandrium to lateral thickenings of dorsal bridge of paramere in the point of connection of *pm db* with gonocoxites (= gonocoxal apodeme) (Figure 6B, C). Lateral thickenings of dorsal bridge of paramere interpreted as gonocoxites according to attachment sites of muscles *M5*.

*Muscles of the hypandrial complex.* Paired long retractors *M1+2* extending from anterior edges of gonocoxites to aedeagal condyle, and also several muscle filaments of *M1+2* extending from anterior edges of gonocoxites to posterior margin of dorsomedial element of paramere *pm dme* (Figure 6C). Paired long *M23* extending from posterior edge of ejaculatory apodeme (dorsally) to membrane near aedeagus (Figure 6A). Paired wide protractors *M30* extending from anterior half of ejaculatory apodeme (laterally) to anterior margin of gonocoxites (= gonocoxal apodeme) (Figure 6A, C). Paired wide retractors *M31* extending from posterolateral part of ejaculatory apodeme to medial anterolateral projections of dorsal bridge *pm db* (Figure 6C). Paired long wide muscles *M27* extending from anterior part of gonocoxites to ligament near anterior margin of gonostyli (Figure 6C). Paired long wide *M28* extending from anteromedial part of gonocoxites to condyle of gonostyli (Figure 6C).

*Muscles of the epandrial complex.* Two pairs of *M3* muscles; *M3^1^* extending from lateral parts of epandrium to anterolateral parts of hypoproct (X sternite); *M3^2^* extending from posterolateral parts of epandrium to membrane between epandrium and cercus (Figure 6D, E). Paired thin *M7* extending from membrane near hypoproct to anterior lobe of cercus (Figure 6E). Hypoproct apparently partly membranous, according to site of attachment of muscles *M3^2^* and *M7*.

## Discussion

### Comparison of *Araucoderus* and *Nothoderus* sclerites and musculature

The sclerites and musculature of *Araucoderus* and *Nothoderus* are similar, but differ in several features. The overall pattern of male terminalia of both genera is similar to most of the Southern Hemisphere genera, with simple curved gonostyli, lobe-like setose parameres, and setose cerci inconspicuous under the epandrium. The terminalia of *Nothoderus* are distinct from genitalia of *Araucoderus* in shape of epandrium with tiny concave median lobe, absence of sclerotized protruding parameral elements and simple aedeagus. However, these differences in structure of terminalia in *Araucoderus* and *Nothoderus* are not associated with differences in musculature. Thus, in *Araucoderus* parameral elements are more diverse and developed and the structure of trifid and simple aedeagus is different; but the aedeagal muscles *M1+2* and ejaculatory muscles *M23*, *M30* and *M31* look very similar in both genera.

We have found different degrees of sclerotization of the hypoproct in *Araucoderus* and *Nothoderus*, and correlated differences in muscles of the epandrial complex. The hypoproct is recessed deeply within the genitalia; its location is possibly the reason why the hypoproct was not mentioned by some previous researchers (Krzemiński and Judd 1997). *Araucoderus* is characterized by the well-developed hypoproct connection to the wide muscles *M3*, whereas the hypoproct of *Nothoderus* is partly membranous, according to the site of attachment of muscles *M3^2^* and *M7* (both attached not to hypoproct but to a membrane near it). The weakening and division of muscles *M3* into two pairs of muscles in *Nothoderus* is probably associated with this membranization.

One interesting difference associated with rotation was found in the abdominal structure. *Nothoderus* is characterized by the 180° rotation of segments VII–IX, of the six specimens examined, four males demonstrated clockwise rotation (two of them only 160°; Figure 5) and two counterclockwise rotation; one of them had its terminalia rotated only 90°. In other families of nematocerous Diptera, this rotation often occurs several days after emergence, so probably it was a young male captured during its first days of activity (for details see Lukashevich 2018).

*Araucoderus* is also characterized by 180° rotation of segments VII–IX. However, of the 12 males of *Araucoderus* examined by us, seven had partially rotated terminalia (about 90°) and five males had terminalia with 180° rotation; eight specimens had clockwise rotation and 4 males had counterclockwise rotation (such males are illustrated in Figures 1A, 2). Three males were collected by one of us during one hour on the same beach (Lenca River, Alerce Andino NP, 12.i.2015); one male had terminalia with 90° clockwise rotation, two others with 90° and 180° counterclockwise. Madriz and Courtney (2016, fig. 1) also recorded 90° and 180° rotation for *Araucoderusgloriosus* and figured clockwise rotation on the total view without any mention of reverse cases. The difference of rotation direction is common among nematocerous Diptera; e.g. the rotated genitalia were randomly oriented and similar numbers of clockwise and counterclockwise events were observed in sufficiently large samples within the same species in Culicidae and Psychodidae (Chevone and Richards 1976, Curler et al. 2015, Votypka et al. 2015). We believe that Tanyderidae are not exceptions and both genera have similar rotated terminalia with 180° (and 90° as an intermadiate stage), rotation may be either clockwise or counterclockwise in a 1:1 ratio. However, in spite of the similar results, this rotation is carried out in different ways in *Araucoderus* and *Nothoderus*.

*Araucoderus* is characterized by the narrow segment VIII, but not reduction of the separate sternite and tergite VIII. *Nothoderus* has segment VIII strongly reduced, with tergite VIII totally membranous and sternite VIII as a narrow sclerite. Accordingly, attachment sites and thickness of pregenital muscles are greatly different between the genera: reduction of muscles *M19* and unpaired *M18* was found in *Nothoderus*, whereas *Araucoderus* has paired symmetrical *M18* and *M19* (see below, Table 1).

The attachment sites of muscles *ISM7* and *M18* confirm the origin of sternite VIII, and the attachment sites of muscles *ITM7* confirm the entire membranization of tergite VIII of *Nothoderus* (Figure 5). Moreover, attachment sites of muscles *M18* (between sternites VIII and IX) confirm the homology of the hypandrium (sternite IX) as the narrowly sclerotized stripe between gonocoxite bases of both *Araucoderus* and *Nothoderus* (Figures 1E, 4A, 5B). Localization of these muscle attachment sites is reliable evidence of the hypandrial origin of the sclerites, as it was revealed by us for DipteraCyclorrhapha (Galinskaya and Ovtshinnikova 2015a, b, Ovtshinnikova and Galinskaya 2016a). Merging of dorsal bridge with gonocoxites is confirming according to attachment sites of muscles *M5* (*M5* connecting anterolateral parts of epandrium to lateral thickenings of dorsal bridge of paramere in the point of connection of *pm db* with gonocoxites) (Figure 6B, C).

The reduction and asymmetry of segment VIII is noted for Tanyderidae for the first time. Such reduction of segment VIII contradicts Alexander’s opinion of *Nothoderus* as “the most generalized of the living Tanyderidae” (Alexander 1928). Moreover, the free tip of Sc which was the reason for his conclusion, is not yet known in the fossil record (pers. obs. EDL), so we assume that it is also a derived character.

One more interesting difference was found in the structure of abdominal intersegmental muscles of VII sclerites. These muscles are asymmetrical and the sternal muscles *ISM7* are paired in both genera, whereas the tergal muscles *ITM7* are paired in *Araucoderus* but in *Nothoderus* only one unpaired *ITM7* was found (Table 1; Figures 2, 5). The position of these muscles as well as their symmetry in Ptychopteridae (the family without rotation of male terminalia) indicates that they probably provide the rotational force for inversion of the terminalia in Tanyderidae and Psychodidae.

### Relationship of Tanyderidae and their supposed relatives: male terminalia evidences

As it was discussed in the introduction, different authors cluster Tanyderidae with Blephariceridae, Ptychopteridae or Psychodidae. Data on skeleton and musculature of male terminalia in Tanyderidae, Psychodidae, Blephariceridae and Ptychopteridae are compared in Tables 1, 2 and illustrate the noticeable peculiarity of Tanyderidae.

Among these families only Tanyderidae are characterized by ejaculator muscles *M23* (in addition to muscles *M30* and *M31*, present in other families). Muscles *M23* connect only sclerites or membranes of aedeagal complex consisting of ejaculatory apodeme and aedeagus. Up to now these muscles were recorded in the only one nematocerous family, Trichoceridae, and in Brachycera (as *M32* in Ovtshinnikova 1989). Based on our data one can suppose that muscles *M23* are present in the groundplan of the Diptera.

It can be assumed that muscles *M30* and *M31* of Tanyderidae, attached to different parts of the ejaculatory apodeme, have the opposite functions of muscles *M30* and *M31* of Blephariceridae and Bibionidae and most of Brachycera, muscles *M31* of Psychodidae and Ptychopteridae. Muscles *M30* of Tanyderidae are, probably, protractors, muscles *M31* of Tanyderidae are, probably, retractors as in Trichoceridae (Ovtshinnikova 1989). Protractor muscles in these families are usually wide and fan-shaped, their attachment sites occupy a wide surface of the ejaculatory apodeme, regardless of the origin of the muscles (muscles *M30* or *M31*). At the same time, the other side of the protractor muscles of Tanyderidae is attached to the gonocoxites, therefore the muscles are designated as *M30*, as well as in the most other families (including Brachycera). Retractors are narrower and often connect to the base of the ejaculatory apodeme. In the Tanyderidae these muscles are connected to parameres, and are designated as *M31*, in other families these muscles are associated with gonocoxite apodemes or gonocoxites and are designated as *M30*. Therefore, in this case we assume a complete changing of the functions of these paired antagonist muscles.

Only Tanyderidae are characterized by the merging of aedeagal muscles *M1* and *M2*. Psychodidae, Blephariceridae, and Ptychopteridae are characterized by separate aedeagal muscles: the protractors *M1* and retractors of aedeagus *M2* and two pairs of aedeagal muscles are part of the dipteran groundplan (one pair *M1* and one pair of *M2*). Tanyderidae are characterized only by retractors *M1+2* that should lead to changing in mechanism of aedeagus functioning.

The examined tanyderids are characterized by muscles *M1+2* connecting gonocoxites partly to the aedeagus, partly to the dorsomedial element of the parameres *pm dme*, and by muscles *M31* connecting posterior part of ejaculatory apodeme to medial part of dorsal bridge of paramere *pm db*. Tanyderidae as well as Psychodidae and Blephariceridae are characterized by a sclerotized bridge forming through medial merging of parameres and “connecting the gonocoxites dorsally via the gonocoxal apodemes” (Sinclair 2000). It can be the initial stage of the aedeagal sheath forming: the fused parameres with attached powerful muscles provides protection of the aedeagus and increases the pulling of the aedeagus during copulation, whereas the aedeagus remains well sclerotized and independent. Therefore, the presence of the bridge could be a derived state for Tanyderidae, Psychodidae and Blephariceridae, however, the parameres are fused dorsally in many lineages, e.g. in Trichoceridae, Limoniidae and Axymyiidae (Wood 1991, Sinclair 2000; Paramonov 2004; Sinclair et al. 2013) and it is possible that the dorsal bridge connecting the gonocoxites dorsally via the gonocoxal apodemes is a part of the dipteran groundplan.

Blephariceridae are distinct from Tanyderidae in tergosternal muscles *M5* divided into two pairs and the absence of terminalia rotation (Table 2). Within the discussed families only Blephariceridae and Tanyderidae are characterized by a trifid aedeagus with three slender filaments, each with separate openings. There are currently two opinions on the number of aedeagal openings in the dipteran groundplan: three (based on basal number of spermathecae; e.g. Downes 1968, Petersen et al. 2010) or one (based on outgroup comparison, e.g. Wood 1991, Ribeiro 2008). Following the view of Wood (1991) we consider the trifid aedeagus as an apomorphic state, which evolved independently and repeatedly in different families (in Cylindrotomidae and some Brachycera besides Tanyderidae and Blephariceridae). It is obvious that a shift from single to multiple openings has occurred multiple times (Wood 1991) and Tanyderidae is one more example in this sequence (with the single opening in *Nothoderus* and three phallothremata in *Araucoderus*). Taking into account a bifid aedeagus (*Peringueyomyina* and several extinct genera, Madriz 2017, Lukashevich 2018), we can infer that the disparity of the aedeagus structure in tanyderids appears to be the most extreme in the order, which makes it impossible to draw any conclusions on relatives in the absence of an established phylogeny of Tanyderidae.

**Table 2.** Characters of male terminalia of Tanyderidae, Psychodidae, Blephariceridae and Ptychopteridae, discussed in the text (1 – after Just 1973; 2 – after Zwick 1977).

	**Rotation**	**Trifid aedeagus**	**Sperm pump hypertrophied**	**Aedeagal muscles**		**Tergosternal muscles M5**	**Ejaculator muscles**			**Gonocoxal muscles M33**
				**M1**	**M2**		**M23**	**M30**	**M31**	
** Tanyderidae **										
* Araucoderus gloriosus *	+	+	–	one pair		one pair	one pair	one pair	one pair	–
* Nothoderus australiensis *	+	–	–	one pair		one pair	one pair	one pair	one pair	–
** Psychodidae **										
* Pneumia palustris * **^1^**	+	–	–	one pair	one pair	two pairs	–	one pair	one pair	–
* Phlebotomus garnhami * **^1^**	+	–	–	one pair	one pair	–	–	–	two pairs	–
** Blephariceridae **										
* Edwardsina gigantea * **^2^**	–	+	–	one pairs	one pair	two pairs	–	one pair	two pairs	–
** Ptychopteridae **										
* Ptychoptera lacustris * **^1^**	–	–	+	four pairs	two pairs	one pair	–	–	one pair	one pair

Ptychopteridae are also distinct from Tanyderidae in the absence of terminalia rotation, and share plesiomorphic characters only, such as tergosternal muscles *M5* not divided (Table 2). Ejaculatory-aedeagal complex of Ptychopteridae is very specialized, e.g. the spherical sperm pump is hypertrophied as compared with other dipteran families (including the discussed ones), with only one pair of ejaculator muscles *M31* and with six pairs of aedeagal muscles *M1^1–3^* and *M2^1–2^* (Tanyderidaeare characterized by one pair of merged *M1+2*). Ptychopteridae are characterized by one pair of hypandrial muscles of gonocoxite *M33* which is a feature of the groundplan of the Diptera (Paramonov 2004), whereas in Tanyderidae, Psychodidae, and Blephariceridae*M33* is absent. We did not find obvious male terminalia synapomorphies grouping Tanyderidae with Ptychopteridae.

Psychodidae is the single family under discussion with male terminalia rotation (for details see in Lukashevich 2018) although genera with unrotated male terminalia are known in Sycoracinae and Horaiellinae (Duckhouse 1972, Curler and Priyadarsanan 2015). In *Phlebotomus*, the rotation begins with segment VII as in both tanyderids described here. The genus has asymmetrically paired abdominal intersegmental muscles of VII sclerites as in *Araucoderus* and posterior sites of attachment of muscles *ITM7* and *ISM7* are slightly moved clockwise (Just 1973), similar to *Nothoderus*, but unpaired abdominal or pregenital muscles were not found in Psychodidae. We believe that Tanyderidae is closely related to Psychodidae, but the musculature of male terminalia offers rather little confirmation of this relatedness: we did not find any important similarities in the musculature of Psychodidae and Tanyderidae. The absence of *M33* in both families is not such an important similarity, although *M33* was found in Ptychopteridae, Trichoceridae, Pediciidae, Tipulidae and in some Bibionomorpha and Brachycera and is a considered part of the dipteran groundplan (Ovtshinnikova 1989, Paramonov 2004). However, *M33* is also absent in Limoniidae and some Bibionidae (Ovtshinnikova 1989, Paramonov 2004), so its loss seems to have occurred several times independently.

It is worth noting that Psychodidae are extremely diverse and the scarce data on their musculature confirms this diversity: e.g., Psychodinae are characterized by tergosternal muscles *M5* divided into two pairs, whereas Phlebotominae are characterized by absence of muscles *M5* (Just 1973, Jobling 1987, figure 111). At the same time, muscles *M5* are usually very stable within different families of Diptera (Ovtshinnikova 1989, 1993, 2000); e.g., Limoniidae has *M5* but *M5* is absent in Tipulidae and this character is considered an autapomorphy of the latter family by Paramonov (2004). Due to such diversity within Psychodidae, it is impossible to make serious conclusions based on only two examined derived genera, *Pneumia* and *Phlebotomus*, while data on more basal subfamilies are absent. Bruchomyiinae has been referred to as the sister group to the remaining Psychodidae by some authors (Quate and Alexander 2000) and earlier this subfamily was even included in Tanyderidae (Alexander 1927), whereas other authors hypothesized a more basal position for Sycoracinae and Horaiellinae (Curler and Moulton 2012), but the musculature of these subfamilies has not been studied.

## Conclusions

The Tanyderidae are characterized by very specialized sclerites and muscles of male terminalia; these structures provide no evidence of relationship with previously studied members of Psychodidae, Blephariceridae and Ptychopteridae. Within these three families, only Psychodidae have obligatory 180° male terminalia rotation and only Blephariceridae have a trifid aedeagus. Although our initial hypothesis was the similarity of Tanyderidae and Psychodidae and we looked for evidence using the analysis of musculature characters that had not previously been investigated; the musculature of male terminalia offers little confirmation of this relatedness. The absence of evidence is probably connected with the absence of data on musculature of the primitive psychodid subfamilies Bruchomyiinae, Sycoracinae and Horaiellinae.

## Supplementary Material

XML Treatment for Araucoderus gloriosus

XML Treatment for Nothoderus australiensis
